# Effects of Online Motor Control Exercises on Employees of Companies

**DOI:** 10.7759/cureus.84994

**Published:** 2025-05-28

**Authors:** Tsuyoshi Morito, Nahomi Tsujioka, Rihoko Kawamuro, Ayane Sugiura, Hinako Ezaki, Mari Chiba, Yuzuki Kurishima, Michihiko Nishina, Koji Kaneoka

**Affiliations:** 1 Sport Sciences, Waseda University, Shinjuku-ku, JPN; 2 Therapeutics, STUDIO MUNI, Shinagawa-ku, JPN; 3 Therapeutics, Rihoko Yoga, Fukuoka City, JPN; 4 Sports Sciences, Waseda University, Shinjuku-ku, JPN; 5 Orthopaedics, Coretrim Station, Shibuya-ku, JPN

**Keywords:** company employees, low back pain, motor control exercise, occupational health, online session, physical functions

## Abstract

This study aimed to investigate the effects of online instructions regarding whole-body motor control exercises (MCEx) on company employees. A total of 30 participants (average age of 47 years) were recruited for this study from two companies. One instructor per company instructed groups of approximately 15 participants each on MCEx online for about 30 minutes for 12 weeks. The KOJI AWARENESS^TM^ (KA; a self-rated whole-body movement assessment system), the degree of pain in each joint (numeric rating scale or NRS), presenteeism, and the 36-Item Short Form Survey (SF-36) scores were assessed before and after the intervention and compared. Twenty-five of the 30 participants were included in the analysis. The NRS for low back pain significantly improved from 3.6±2.5 before the intervention to 2.5±2.3 after the intervention (*p*<0.05). The SF-36, which assesses quality of life, showed statistically significant improvements in physical function (pre: 86.6±11.0; post: 92.0±6.2) and vitality (pre: 49.8±20.2; post 57.3±21.1). Presenteeism also improved significantly, from 21.1±18.4 % to 13.4±13.1%. In addition, KA scores increased significantly from 37.6±8.0 to 43.3±6.7 after the intervention (*p*<0.05). Improving the motor control function of employees within enterprises through online MCEx may enhance motor unit function, reduce complaints, and contribute to greater productivity.

## Introduction

Health management in companies is flourishing. Health management refers to the strategic implementation of health management for employees from a managerial perspective. Based on corporate philosophy, investment in the health of employees and others should increase employee and organizational vitality, including increasing productivity, which in turn, leads to improved business and stock price performances. A study of 12,350 workers in Japan quantified presenteeism, absenteeism, and medical costs and reported that economic losses due to presenteeism were the highest at 64% of the total [1]. In addition, national data revealed occupational differences in health-related productivity loss. Presenteeism is notably higher among healthcare, education, and service workers, whereas absenteeism tends to be more frequent in physically demanding jobs such as construction and transport [2]. A nationwide study also reported that the prevalence of self-reported low back pain (LBP) was 14.2% among women and 11.7% among men, with higher rates among non-regular and agricultural workers [3]. Furthermore, the top three causes of reduced productivity among workers were neck pain/shoulder pain, lack of sleep, and LBP. In particular, chronic low back pain (LBP) has been associated with various aspects of work functioning impairment, including "time management," "concentration/interpersonal relationships," and "work results," as assessed using the Work Functioning Impairment Scale [4]. Furthermore, several recent studies have shown that the COVID-19 pandemic, particularly due to the shift to remote working, has led to a marked decrease in physical activity and an increase in sedentary behavior. For example, a study by the Estudo Longitudinal de Saúde do Adulto (ELSA)-Brasil project reported that telecommuters during the pandemic had a median total sedentary time exceeding eight hours per day, which was significantly higher than that of on-site workers [5]. Similarly, in a review of global data, 76% of the studies reported a decrease in physical activity levels during the pandemic, with increased sedentary behavior being a prominent concern [6]. Therefore, interventions are required for the current company workers.

Exercise therapy is generally recommended to improve LBP and shoulder stiffness, which are common among workers. The usefulness of motor control exercises (MCEx) has been previously reported. MCEx are exercises aimed at restoring the coordinated and efficient use of the muscles that control and support the spine [7], and have been shown to be effective in reducing pain intensity and improving function in patients with chronic non-specific LBP. In particular, these effects have been shown to persist over the short, medium, and long term, especially when compared with treatments such as manual therapy and other types of exercises [8]. Its benefits for healthy people include improved posture and performance. Leading swimmers perform MCEx, which involves stretching to increase joint mobility and optimize the use of the body, during long swim practice sessions prior to rigorous swimming practice, thereby preventing injury and achieving a higher performance [9]. Similarly, performing these exercises to condition the body before performing any kind of exertion or work in companies may increase work efficiency and prevent musculoskeletal disorders. However, few studies have implemented MCEx among employees or tested the effects of the intervention. Therefore, this study aimed to determine the impact of MCEx on the physical function and productivity of workers in enterprises.

In recent years, especially following the pandemic, the online landscape has expanded significantly, demonstrating the advantages of online group exercises [10]. We hypothesized that online MCEx for company employees would improve their physical functioning and increase their productivity.

## Materials and methods

Participants

The Japan Sports Agency commissioned this study, and those who requested to participate in response to the announcement were enrolled in this single-arm study. A total of 30 employees applied to participate in the online MCEx sessions, including 26 from Company A and four from Company B. Participants from both companies were engaged in desk-based work. The sample size was set as effect size of f=0.4, α=0.05, and power=0.8 to 27. The inclusion criteria were adult men and women aged 18 years or older, and the exclusion criteria were current and preexisting medical conditions and the inability to obtain consent from their doctor to participate in the study. Participants with shoulder joint pain, LBP and neck pain were included. Informed consent was obtained from each participant. This study was conducted in accordance with the Declaration of Helsinki after obtaining approval from the Ethics Review Committee on Research with Human Subjects of Waseda University (approval number: 2022-538). The study was registered with the University Hospital Medical Information Network (UMIN).

Pre- and post-intervention data

Data gathered from the participants included basic information (age, sex) and data on the presence of LBP, shoulder pain, and neck pain the location and severity of pain (NRS), their SF-36 [11] score, and presenteeism assessed using the Single-Item Presenteeism Question (SPQ) [12] were collected. In the SPQ, the respondents rated their own performance between 1% and 100%, with 100% defined as their best performance in the absence of illness or injury. The difference between the respondent’s rating and 100 was defined as “SPQ presenteeism,” meaning that the higher the SPQ presenteeism, the greater the degree of productivity loss. The NRS score for pain in each joint was indicated on a scale of 0 to 10. The SF-36 score on the quality of health status is presented as a 100% conversion value for the following scales: physical functioning, daily role functioning (physical), body pain, overall sense of health, vitality, social functioning, daily role functioning (mental), and mental health. Physical function was measured using the KOJI AWARENESS^TM^ (KA) [13], and as in previous studies, it was indicated on a 50-point scale. All measurements were taken before and after the three-month intervention.

Implementation of the intervention (three months) 

**Figure 1:**
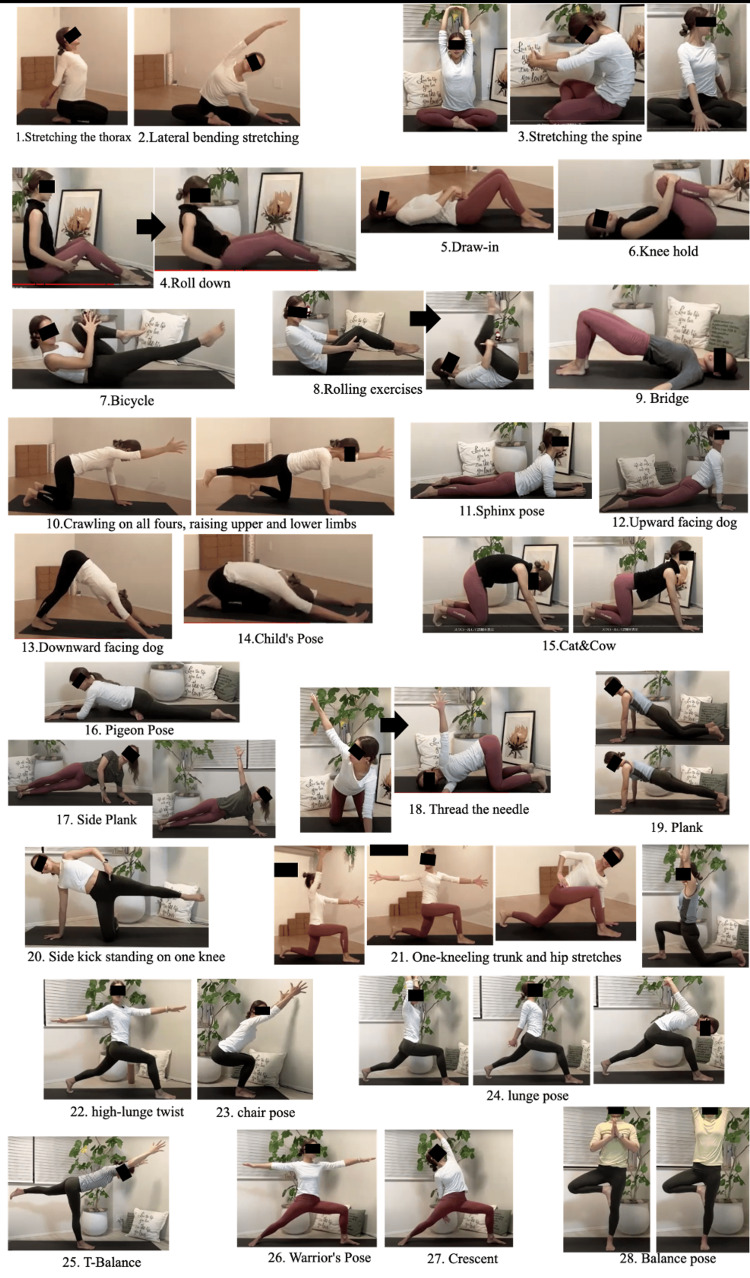
The list of motor control exercises The motor control exercises (MCEx) were performed by two certified instructors with seven and four years of experience, respectively, for 30 minutes per session, two days a week for a total of 12 weeks. The MCEx were demonstrated online and were based on yoga and Pilates. Participants were divided into groups of up to 15, and an instructor checked that the exercises were performed correctly. The exercises were repeated approximately two to three times. The instructional content was changed every time. The basic exercises were first performed in the supine position or on all-fours and then gradually modified to be performed in the standing position.

The MCEx sessions were conducted by two qualified instructors with seven and four years of experience, respectively. They were for 30 minutes each and were conducted twice a week for a total of 12 weeks. Both instructors had the same teaching credentials, and the frequency and duration of their correspondence was the same. Zoom (Zoom Video Communications Inc., CA, USA) was used to conduct the MCEx online. The MCEx sessions were based on yoga and Pilates, and started at 8:00 or 9:00 p.m. outside of work hours. Participants participated from their home computers or smartphones. The sessions were recorded, and the participants could also watch the on-demand video. The MCEx were performed to improve the motor control function of the spine; the mobility of the spine, thorax, and hip joints; and the function of the shoulder girdle. The participants were divided into groups of up to 15, with the instructor checking whether the exercises were performed correctly. Approximately two or three repetitions of the exercises were performed. Demonstrations of the MCEx performed were also delivered via videos, and participants were encouraged to perform them daily whenever possible (Figure 1 and Table 1).

The instructions were changed from time to time. The basic exercises were first performed in the supine position or with the participant on all-fours and then gradually modified till they were performed in the standing position.

**Table 1:** Motor control exercises

Exercise name	Purpose
1. Stretching the thorax	To improve the mobility of the shoulder joint and the thorax
2. Lateral bending and stretching	To improve the mobility of the trunk and the thorax and improve the motor control function of the spine
3. Stretching the spine	To improve the mobility of the lumbar spine, neck, and shoulder girdle
4. Roll-down	To improve the mobility of the spinal column and the motor control function of the spine
5. Draw-in	To activate and improve the function of the transversus abdominis muscles
6. Knee hold	To improve the mobility of the lumbar spine
7. Bicycle	To improve the motor control function of the trunk and the lower limbs
8. Rolling exercises	To improve spinal mobility and its motor control function
9. Bridge	To improve hip strength and the motor control function of the spine
10. Crawling on all fours and raising the upper and lower limbs	To improve hip mobility, trunk and limb coordination, and the motor control function of the spinal column and the pelvic girdle
11. Sphinx pose	To improve the mobility of the thoracic spine and the motor control function of the spine
12. Upward-facing dog (Up-dog)	To improve the flexibility of the chest and the shoulders, strengthen the core and arm muscles, improve the motor control function of the trunk, and improve posture
13. Downward-facing dog (Down-dog)	To improve the mobility of the spine and hip joints and the motor control function of the trunk, lower limbs, and upper limbs
14. Child’s pose	To improve the mobility of the lumbar spine and for relaxation
15. Cat and cow	To improve the mobility of the spinal column, function of the shoulder girdle, and motor control function of the spinal column
16. Pigeon pose	To improve hip mobility
17. Side plank	To improve the motor control function of the trunk, lower limbs, and upper limbs
18. Thread the needle	To improve the mobility of the thoracic spine and the spinal motor control function
19. Plank	To increase the strength of the trunk muscles and improve the motor control of the trunk
20. Side kick standing on one knee	To increase the strength of the trunk muscles and improve the motor control of the trunk and lower limbs
21. One-kneeling trunk and hip stretches	To improve the mobility of the spine and hip joints and the motor control functions of the trunk, lower limbs, and upper limbs
22. High-lunge twist	To improve the mobility of the spine and the hip joint, the motor control function of the trunk, lower and upper limbs, and improve balance
23. Chair pose	To improve the motor control function of the trunk, lower limbs, and upper limbs and improve the muscle strength of the lower limbs
24. Lunge pose	To improve the motor control function of the trunk, lower limbs, and upper limbs and improve the muscle strength of the lower limbs
25. T-balance	To improve mobility of the spine and hip joint, the motor control function of the trunk, lower limbs, and upper limbs, and balance
26. Warrior’s pose	To improve mobility of the spine and hip joint, the motor control function of the trunk, lower limbs, and upper limbs, and balance and posture
27. Crescent	To improve the motor control function of the trunk, lower limbs, and upper limbs and balance and posture.
28. Balance pose	To improve the motor control function of the trunk, lower limbs, and upper limbs, and balance and posture

Statistical analysis

Participants whose questionnaires were available before and after the intervention were considered to have valid data. Those unable to participate in the program for any reason were considered dropouts. Only data obtained before and after the intervention were subjected to the modified intention-to-treat (mITT) analysis, which compared data from all participants before and after the intervention regardless of how often they performed the exercises.

Statistical analyses were performed using IBM SPSS Statistics for Windows, Version 29 (Released 2023; IBM Corp., Armonk, New York, United States). Normality and equal variances of the data were checked using the Shapiro-Wilk and Levene tests, respectively. A corresponding t-test was used to compare each item before and after the intervention depending on the normality of the data. The items “Have only LBP,” “Have only shoulder stiffness,” “With both LBP and shoulder stiffness,” and “Neither LBP nor shoulder stiffness” were subjected to a chi-square test before and after the intervention.

The significance level was set at p <0.05. Effect sizes were calculated using Cohen’s d [14] (0.20 to less than 0.50 was defined as a “small effect size,” 0.50 to less than 0.80 as a “medium effect size,” and 0.80 or greater as a “large effect size”). Cramér’s V was used to estimate the effect size for the chi-squared test (values from 0.10 to less than 0.30 were defined as “small effect size,” 0.30 to less than 0.50 as “medium effect size,” and 0.50 or greater as “large effect size”).

## Results

Data from five of the 30 participants were difficult to obtain before the three-month assessment was carried out after the first visit; therefore, 25 were included in the analysis (mean age 48.5±9.8 years, 10 men and 15 women). Of the 25 participants, 18 attended at least once a week, accounting for 72% of the total. Seven of the 25 participants had weeks in which they were unable to attend at least once.

After three months of intervention, the degree of shoulder (pre: 2.3±2.5; post: 2.6±2.3) and neck pain (pre: 3.2±2.6; post: 3.4±2.7), as measured by the NRS, showed no significant change, but the degree of low back pain improved (pre: 3.6±2.5; post: 2.5±2.3; *p*<0.05). SPQ presenteeism improved significantly from 21.1±18.4% before intervention to 13.4±13.1% after intervention (*p*<0.05). In addition, the SF-36, which assesses quality of life, showed statistically significant improvements in two items: physical function (pre: 86.6±11.0; post: 92.0±6.2) and vitality (pre: 49.8±20.2; post: 57.3±21.1; *p*<0.05). Although increases were observed in all eight items, only these two items reached statistical significance. Furthermore, physical function assessed using the KA also significantly improved, with scores increasing from 37.6±8.0 to 43.3±6.7 after the intervention (*p*<0.05). Detailed results are provided in Table 2.

**Table 2:** List of the results pre- and post-intervention *An asterisk symbol indicates *p*<0.05. Effect sizes were calculated using Cohen’s d [14] (0.20 to less than 0.50 was defined as a “small effect size,” 0.50 to less than 0.80 as a “medium effect size,” and 0.80 or greater as a “large effect size”). Cramér’s V was used to estimate the effect size for the chi-squared test (values from 0.10 to less than 0.30 were defined as “small effect size,” 0.30 to less than 0.50 as “medium effect size,” and 0.50 or greater as “large effect size”).

Measurement		Pre-intervention	Post-intervention	Effect size
NRS	Low back pain	3.6±2.5	2.5±2.3	d=0.46*
	Neck pain	3.2±2.6	3.4±2.7	d=0.01
	Shoulder pain	2.3±2.5	2.6±2.3	d=0.12
SF-36	Physical functioning	86.6±11.0	92.0±6.16	d=0.60*
	Role, physical	84.0±19.0	86.0±15.8	d=0.11
	Body pain	66.1±18.8	66.6±21.9	d=0.02
	General health	60.7±15.0	63.0±14.4	d=0.16
	Vitality	49.8±20.2	57.3±21.1	d=0.36*
	Social functioning	87.0±16.4	87.5±15.8	d=0.03
	Role, emotional	79.7±25.2	84.3±20.3	d=0.20
	Mental health	70.8±14.2	71.6±14.7	d=0.06
Single-Item Presenteeism Question (%)		21.1±18.4	13.4±13.1	d=0.48*
KOJI AWARENESS^TM^		37.6±8.0	43.3±6.7	d=0.78*
Have only LBP (n)		2	3	
Have only shoulder stiffness (n)		7	7	V=0.21
With both LBP and shoulder stiffness (n)		12	8	
Neither LBP nor shoulder stiffness (n)		4	7	

## Discussion

Online MCEx for office workers improved some quality-of-life items and physical functioning and reduced LBP and presenteeism. It improves physical function and has been shown to increase muscle activation and coordination, particularly in the trunk and pelvic region. Such improvements have been shown to enhance athletic performance and reduce the risk of injury in healthy individuals [15]. Another study suggested that regular MCEx promote positive neuroplastic changes and the brain’s ability to adapt to new motor tasks and challenges, which may help maintain motor performance and prevent age-related decline in motor function [16]. Regular MCEx may also be beneficial for employees without motor disorders because performing these exercises to improve physical conditioning may increase work efficiency and prevent such disorders.

LBP and stiff shoulders are among the most common complaints of employees in companies, affecting labor productivity and company management. In recent years, with the development of teletherapy and online learning, MCEx has been taught online with good results [10]. Most working individuals have sedentary jobs, and prolonged sitting can lead to LBP and shoulder stiffness. In addition, remote working, partly because of the recent pandemic, has reduced the amount of travel and physical activity [5]. A survey of the average sitting time on weekdays in 20 countries showed that Japan had the highest number of sedentary workers, spending the longest number of hours on sedentary work [17]. The MCEx conducted in this study may have reduced LBP and shoulder stiffness and improved presenteeism. Deep trunk muscle function is important for improving LBP. Previous studies have shown that patients with LBP have reduced function of the deep trunk muscles, such as the transversus abdominis and multifidus muscles [18], and these exercises may have stimulated the activity and improved their function [19]. The ability to move joints under one’s power, the so-called mobility, may have also improved, and the improvement in KA reflects this. The program, which aimed to improve and enhance whole-body motor control function, improved spinal mobility and was particularly effective for participants with LBP.

The SF-36 was also used in this program, with scores on all eight items increasing post-intervention compared to pre-intervention. These increased scores may be related to physical function and ease of movement due to reduced pain. There is consensus on the positive effects of exercise on mental health [20]. Exercising once a week with a trainer or a group member may have positive effects on social functioning [21]. Furthermore, participation in a weekly program provides a “change of pace” and “communication,” as shown in the Health Management Office Effectiveness Model [22], which may have resulted in a synergistic effect. Online MCEx may also achieve similar effects.

In recent years, KA has been measured in healthy people, and it has been shown that the scores decrease, that is, physical function declines, around 50 years of age [23]. Approximately 50 years is also the most common median age for employees in companies and is the so-called prime working age. Measures to improve the conditioning of the musculoskeletal system at or before this age would be effective in improving the workforce and supporting the country’s finances. Furthermore, disorders with the locomotor system are associated with locomotive syndromes and internal medicine disorders. In the future, it will be necessary to cooperate with health insurance associations to promote health checkups that examine the locomotor system of workers for primary prevention of locomotor disorders.

The implementation of online MCEx in industrial settings in Japan may play a vital role in promoting occupational health and well-being, particularly by addressing musculoskeletal discomfort and presenteeism among workers. As Japanese workplaces face aging populations and increased sedentary behavior, such accessible, scalable interventions offer promising potential for integration into company-based health promotion strategies.

This study has several limitations. First, there was no control group, and the participants understood that they were participating in an exercise intervention; therefore, the placebo effect could not be ruled out. Future studies should be conducted using a design in which participants are randomly recruited and targeted. Second, the frequency with which the participants performed the exercises at home could not be ascertained. Participants in this study responded to notices and posters, which may indicate a high level of motivation and a positive attitude towards exercise. Motivation is also important for continuation of exercise, and the contributing factors for the same should be investigated in the future. Third, this study had a limited number of participants and a limited design, making it difficult to generalize the results. In addition to MCEx, it is undeniable that changes in daily activity and sitting time may have influenced the results. However, this study is unique in that it attempts to use MCEx to improve the labor productivity and performance of company workers.

## Conclusions

Online MCEx improved physical function, reduced low back pain, and enhanced vitality and presenteeism among company employees. These results suggest that remote implementation of MCEx is a feasible and effective intervention for promoting musculoskeletal health and productivity in the workplace. Further randomized controlled studies are warranted to validate these findings in a larger population.
